# GnRH agonist treatment for idiopathic central precocious puberty can improve final adult height in Chinese girls

**DOI:** 10.18632/oncotarget.22568

**Published:** 2017-11-20

**Authors:** Yanqin Ying, Jing Tang, Wei Chen, Zemin Cai, Wan Ting Niu

**Affiliations:** ^1^ Department of Pediatrics, Tongji Hospital, Tongji Medical College, Huazhong University of Science and Technology, Wuhan, China; ^2^ Department of Pediatrics, Jingzhou Maternal and Children Health Care Hospital, Jinzhou, China; ^3^ Department of Pediatrics, Sichuan Shuangliu Maternal and Children Health Care Hospital, Chengdu, China; ^4^ Department of Pediatrics, The First Affiliated Hospital of University of South China, Hengyang, China; ^5^ VA Boston Healthcare System, Department of Orthopedics, Brigham and Women's Hospital, Harvard Medical School, Boston, MA, USA

**Keywords:** idiopathic central precocious puberty, gonadotropin-releasing hormone agonist, final adult height

## Abstract

**Object:**

To study the outcomes of GnRHa on final adult height in Chinese idiopathic central precocious puberty (ICPP) girls and the involved factor(s) that can predict height gain.

**Methods:**

We conducted a retrospective analysis on 10 years of data obtained from three clinical hospitals from January 2005 to March 2015, and 101 girls with ICPP, who received GnRHa therapy for more than six months and already reached their adult height were enrolled.

**Results:**

Height, bone age, midparent height, HtSDS, sexual development, therapy duration and predicted adult height(PAH)at start and end of GnRHa, and the final adult height(FAH) were recorded and calculated. Their PAH significantly increased at end of GnRHa (158.4±6.00cm), compared to that at the start of GnRHa(153.1±5.37cm) (*P*<0.001), and their final adult height(157.0±4.82) significantly increased compared to PAH at start of GnRHa(P<0.001). There was no difference between PAH at end of GnRHa and FAH(*P*>0.05). After GnRHa therapy, most of the ICPP girls reached their midparent height compared to that at start of GnRHa(*P*<0.01). FAH was positively correlated with Ht at start, and end of GnRHa, PAH at start and end of GnRHa, and also with midparent height (*R*^*2*^=0.59, 0.74, 0.68, 0.73 and 0.80, *P*<0.001). While FAH was not correlated with the duration of treatment and BA at start of GnRHa(R^2^ = 0.15and 0.1, *P*>0.05). The percentage of adult short stature decreased and those reached midparent height significantly increased after GnRHa therapy, compared to that at start of GnRHa(60.6% vs.30.4% and 67.85% vs. 94.64%, respectively, *P*<0.05).

**Conclusions:**

GnRHa therapy to ICPP girls can effectively achieve the final adult height. After GnRHa therapy, most of these patients reached their midparent height, while few of these patients had an adult short stature.

## INTRODUCTION

Idiopathic central precocious puberty (ICPP) is a common disease in children. In China, it is defined as a condition caused by the initiation of hypothalamic-pituitary-gonadal axis, and the development of secondary sexual characteristics that appears in girls before eight years of age or in boys before nine years of age. This development progresses according to the normal process of sexual development, and the cause of central precocious puberty (CPP) could not be determined in clinic. This disease mainly occurs in girls, and the male-to-female incidence ratio is approximately 1:10 [1]. Effects of CPP on girls mainly include the following: increased sex hormone levels induce the early closure of bone age (BA), and result in the impairment of adult height (AH), early menarche and related psychosocial problems [2]. GnRHa is the main drug used in the treatment of ICPP. However, this drug is expensive, and its cost is particularly high when combined with rhGH. In addition, improvements in final adult height (FAH) in CPP greatly varies [3–5].

China has a large population, and the incidence of CPP also continues to increase. At present, no large-sample systematic analysis on the effects of GnRHa treatment for CPP girls on final height in Chinese population has been reported. The purpose of this study was to study the efficacy of GnRHa on the FAH of Chinese ICPP girls, and to analyze the factor(s) involved that can predict the outcomes.

## RESULTS

### Comparison of auxological features of patients among start of GnRHa treatment, end of GnRHa treatment and end of observation

A total of 101 eligible girls with ICPP were enrolled into this study. The data of these girls are shown in Table 1. The average age of onset the secondary sex development was 7.1± 0.75 years. The age of start of GnRHa treatment was 8.4 ± 0.84 years. Furthermore, the BA of initial treatment was 10.6 ± 0.53 years, height was 137.7 ± 6.26 cm, Ht-BA SDS was -1.8 ± 1.06, and PAH at start of treatment was 153.1 ± 5.37 cm. The course of GnRHa treatment was 22.8 ± 9.3 months, the age at the discontinuation of treatment was 10.3 ± 0.50 years, and BA was 11.9 ± 0.21 years. In addition, the height at end of GnRHa treatment was 142.5 ± 6.36 cm, Ht-BA SDS was -1.5 ± 0.84, and PAH was 158.4 ± 6.0 cm. At end of the observation, the FAH of these girls was 157.0 ± 4.82 cm, and Ht-BA SDS was -0.7 ± 0.89.

**Table 1:** Auxological features of patients with ICPP during observation patients during the observation of related indicators

Variable	Start of GnRHa	End of GnRHa	End of observation
CA(yr)	8.4±0.84	10.3±0.50	15.9±1.21
BA(yr)	10.6±0.53	11.9±0.21	-
Ht(cm)	137.7±6.26	142.5±6.36	157.0±4.82
HtSDS-BA	-1.8±1.06	-1.5±0.84	-0.7±0.89
PAH(cm)	153.1±5.37	158.4±6.00	
MPH(cm)			157.7±3.85
FAH(cm)			157.0±4.82

CA: chronological age; BA: bone age; Ht:height; HtSDS-BA: height SDS based on bone age. PAH: predicted adult height; MPH: midparental height; FAH:Final adult height

### GnRHa treatment had significant improvement on FAH

**Figure 1:**
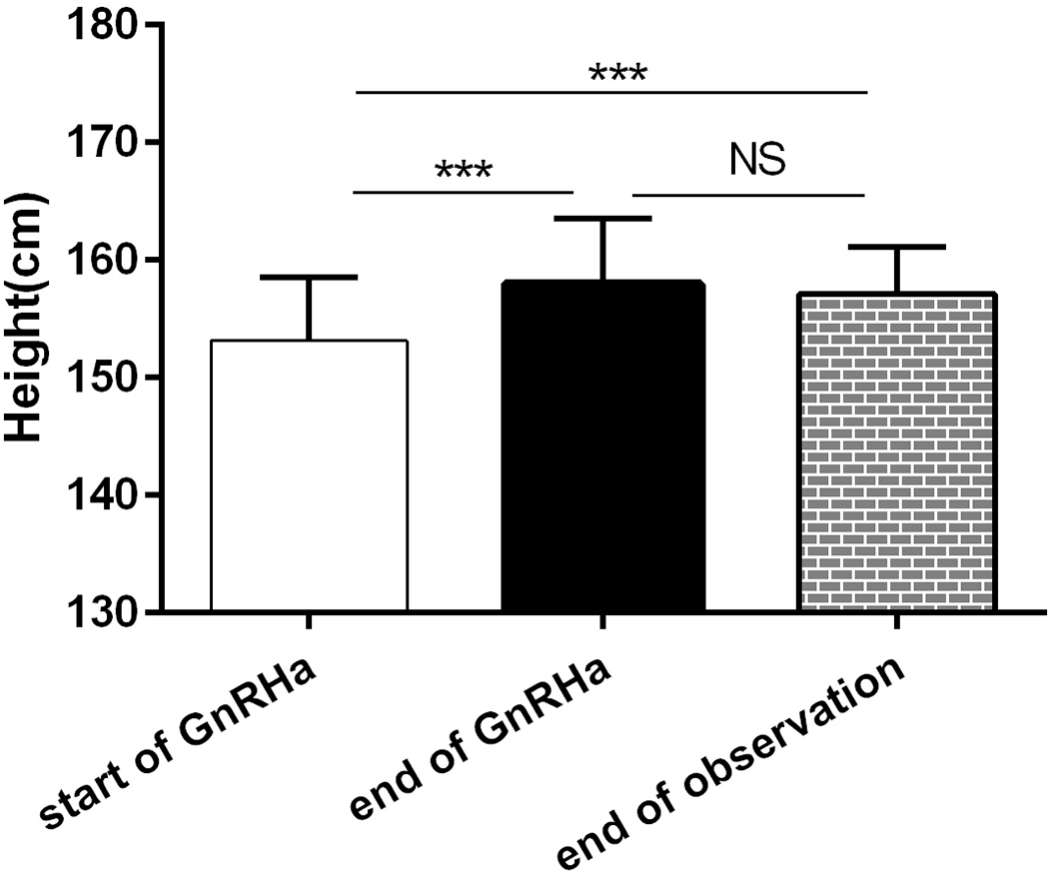
Changes of PAH and AH

To answer the question whether GnRHa can achieve adult height in ICPP girls, we calculated the PAH according to the Bayley-Pinneau method. Differences in PAH and FAH among the three time points (the start of treatment, the end of treatment and the end of observation) were statistically significant (P<0.001), see Figure 1. The PAH increased significantly after GnRHa treatment(158.4±6.00 cm at end of GnRHa vs. 153.1±5.37cm at start of GnRHa, P<0.001). The difference between FAH at end of observation and PAH at end of treatment was not statistically significant (P>0.05). The net height gain after GnRHa treatment was almost 4cm.

### The percentage of adult short stature according to normal adult female height distribution decreased significantly after GnRHa treatment

**Figure 2:**
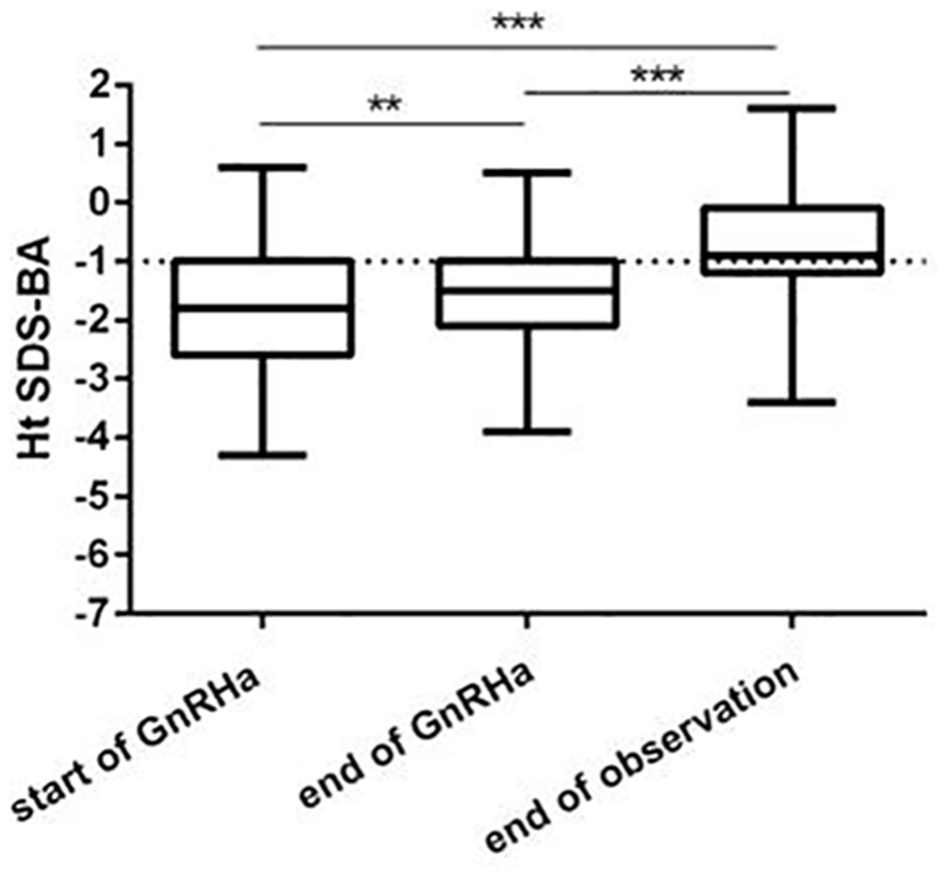
Ht SDS-BA changes during GnRHa therapy and at AH

Ht-BA SDS gradually increased during the treatment(P<0.001). This was significantly increased at end of treatment than that at start of treatment (P<0.01), and Ht-BA SDS was significantly increased at end of observation than that at end of treatment (P<0.001), Figure 2.

**Figure 3:**
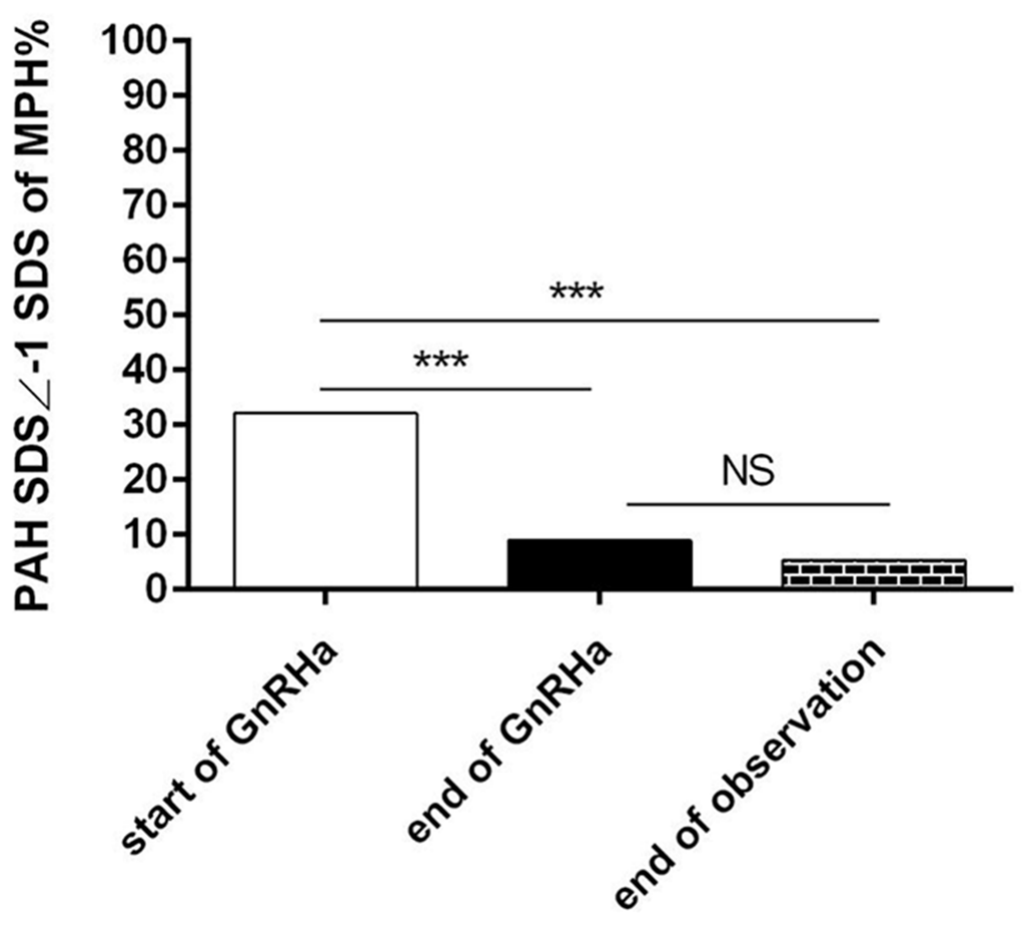
No-reaching normal adult height distribution according to MPH

Since the enrolled patients may not came from the balanced cohort, we took another marker to access whether GnRHa could increase the height gain as PAH SDS based on MPH. We used PAH below -1SDS of MPH as no-reaching their normal height. The percentage of patients whose height did not reach the value of -1MPHSDS was significantly higher at start of treatment (32.14%) than that at end of treatment (8.92%, P<0.001) and at end of observation (5.36%, P<0.001), and the difference between the end of treatment and the end of observation was not statistically significant (P>0.05), Figure 3. This also means that GnRHa therapy can achieve adult height. Figure 3.

**Figure 4:**
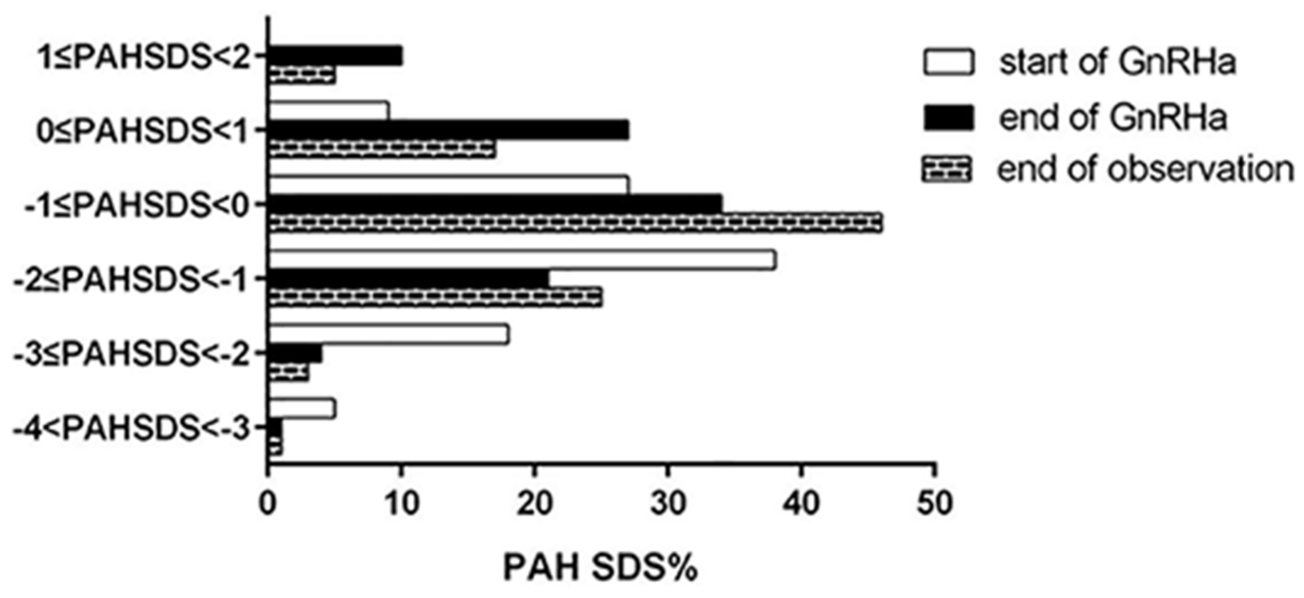
PAH SDS and FAH SDS distribution

As the heterogeneity of height, we compared the percentage of adult short stature or PAH according the normal adult female height distribution and took -1SDS of female adult height as the cut-off value of short stature. The distribution of PAH SDS during treatment is shown in Figure 4. At start of GnRHa treatment, all patients had PAH below 1SDS, and PAH between 0 SDS and 1SDS was only 9.8%, while the percentage below -1SDS was 90.2%. Most of patients had PAH SDS between -1 to -2 SDS(38.4%) and -1 to 0 SDS (27.7%). Then at end of GnRHa treatment, the PAH above 1 SDS increased to 12.0%, and PAH SDS between -1SDS and -2SDS was 24.0%. Most patients increased their PAHSDS to -1 to 0SDS and 0 to 1SDS (39.0% and 31.0%, respectively). This data show that GnRHa treatment improved the height gain. Figure 4.

### Multiple factors regression analysis on final adult height

For one ICPP patient, we cannot tell who will get better height gain or not, or which factors can indicate better final height. To answer these questions, we performed multiple factors regression analysis on final adult height. Related factors that affect FAH were analyzed. It was found that FAH was positively correlated with height at start of treatment, PAH at start of treatment, height at end of treatment, PAH at end of treatment and MPH. However, FAH was not significantly correlated with the age at onset, and the age and BA at start of treatment and duration of treatment (Table 2).

**Table 2:** Multiple regression analysis on FAH

Viriable	Regression coefficient	P-value
CA at onset	0.005	0.48
CA at start of GnRHa(yr)	0.03	0.56
BA at start of GnRHa(yr)	0.1	0.25
Ht at start of GnRHa(cm)	0.59	<0.001
PAH at start of GnRHa(cm)	0.68	<0.001
Ht at end of GnRHa(cm)	0.74	<0.001
PAH at end of GnRHa(cm)	0.73	<0.001
MPH(cm)	0.80	<0.001
Duration of treatment(mo)	0.15	0.85

## DISCUSSION

ICPP mainly occurs in girls, and In 2010, the prevalence of ICPP was 55.9 per 100,000 girls in Asia with annual incidence of CPP ranging from 3.3 to 50.4 per 100,000 [6]. GnRHa is the first choice of drugs for the treatment of CPP, which has been used in clinic for more than 30 years. GnRHa can arrest pubertal progression, and decrease linear growth and skeletal maturation, and consequently should improve FAH and alleviate the psychological problems [7]. However, there were many clinical reports about the outcomes in girls adult heigh, the outcomes varies. several studies reported that GnRHa might also decrease height velocity below the normal range even impaired FAH [8]. The main reason is that in different literatures, the beginning time of treatment of CPP and the therapy indications were different.

In addition, since the cost of GnRHa treatment is high and the difference in height gain after GnRHa treatment in different girls varies dramatically. Toumba and coworkers reported that girls with CPP reached their target height and had a total height gain of 5 cm. Each centimeter cost about €2700($3500) per patient. During treatment, some children needs to additionally get recombinant human growth hormone (rhGH) injection, according to height and growth rate [9–12]. The cost was higher when combined with rhGH. Therefore, follow-ups for the effects of GnRHa treatment on the FAH and the analysis of related factors were necessary, and these are required for the guidance for clinical therapy.

In this study, data on the effects of GnRHa on FAH in girls with ICPP were collected from a number of hospitals in recent 10 years, and correlation analyses of related factors that affect FAH were conducted. Results revealed that GnRHa treatment can significantly improve FAH in the girls with ICPP. After GnRHa treatment, ICPP girls increased their total height gain of 4cm. This suggests that GnRHa treatment can significantly improves FAH in girls with ICPP [13–15]. We didn't observe the final height of ICPP with growth rate less than 4cm/year during GnRHa treatment because most of them got GnRHa combined with rhGH treatment. Jung reported that their 82 GnRHa treated CPP girls achieved final heights approximately 3.8 cm [16]. In a study by Pasquino et al. in which 87 GnRHa-treated (age, 6.5 years) girls with ICPP (age, 6.8 years), the FAH was 159.8 and the 32 no-treated was 154.4 cm [17].

Another issue of this study is whether GnRHa treatment can prove the patient to reach their MPH. We assumed that when the patient's PAH or FAH was above -1 MPH SDS, the patient got their normal height. Our data showed that by GnRHa treatment, the percentage of patients who Reached their MPH increased. This reveals that GnRHa treatment has improved the height gain of girls. In subgroups, we analyzed PAH SDS and FAHSDS distribution before, after GnRHa treatment and end of the observation. We can see that by GnRHa treatment, most of the PAH SDS were above -1PAH SDS while most of the PAH SDS were less than -1PAH SDS at start of treatment, which means most girls achieved their final height. Few studies have focused on the PAH distribution change pattern.

The analysis of related factors that affect the final height revealed that final height was positively correlated with height at start of treatment, PAH at the start of treatment, height at the end of treatment, PAH at the end of treatment and MPH. However, these factors were not significantly correlated with the age at onset, age and BA at the start of treatment and duration of treatment. The factors predicting the height gain were different from different reports. Partsch et al. reported that initial BA advancement and treatment duration were factors that explained 68% of the variability of Ht gain [18]. Others found that growth rate during GnRHa treatment positively correlated to FAH [17, 19].

In summary, In this study about GnRHa treatment in Chinese ICPP girls, the FAH and PAH were significantly higher than the initial height predictions and PAH SDS distribution changed. Therefore, We can concluded that GnRHa treatment is helpful to improve the growth outcomes in girls with ICPP. After GnRHa therapy, most of these patients reached their midparent height.

## MATERIALS AND METHODS

### Selection criteria for subjects

The data of 101 ICPP girls received treatment in three hospitals from January 2005 to March 2015, and have reached their FAH at present were retrospectively collected for this study.

Inclusion criteria:

- Patients comprised of female children who encountered the development of secondary sexual characteristics before the age of eight and were diagnosed with ICPP after luteinizing hormone releasing hormone (LHRH) stimulation test.
- Patients who received treatment with GnRHa alone for more than 6 months
- Patients who discontinued GnRHa treatment when BA reached 12 years during the course of treatment.
- Patients with growth rate in height >4 cm/year during the GnRHa treatment.
- Patients who were followed-up until they reached their FAHs.

Exclusion criteria:

- Patients with a GnRHa treatment course of <6 months.
- Patients with other organic CPP or peripheral precocious puberty.
- Patients who were combined with other diseases that affect height growth and other systemic diseases.
- Patients with a growth rate in height<4cm/year during the GnRHa treatment or they didn't reach their FAHs.

#### GnRHa treatment and follow-up

All patients were given 3.75 mg/time of triptorelin by subcutaneous injection once every 4-6weeks, or 90-150 μg/(kg time) of diphereline with a maximum dose of 3.75 mg/time by intramuscular injection once every 4 weeks. Half a year after the initiation of treatment, X-ray films were taken to evaluate the progress of BA. Thereafter, X-rays were performed once a year, and height and weight measurements, secondary sex development and gonadal hormone levels (LH, FSH, and E2) were assessed once every three months. GnRHa was discontinued when BA reached 12 years. Patients were followed-up once a year after the discontinuation of treatment, X-ray films were taken, and height and weight were recorded. The follow-up ended when FAH was reached.

#### Determination of FAH

AH or FAH is considered to be achieved when BA was ≥15 years or the annual growth rate was <2 cm/year [20].

#### Evaluation methods

(1) BA: BA was determined using the Greulich-Pyle standard chart. (2) Predicted adult height (PAH): The Bayley-Pinneau method (B-P method) was used with the table of prediction of the adult heights for girls whose BAs are greater than their ages [21, 22]. (3) Height-bone age standard deviation score (Ht-BA SDS) method: The Ht-BA SDS was calculated based on BA, with reference to the 1995 Nine-City Urban Physical Examination Data in China. (4) Midparental height (MPH) or target height (TH): The TH of the patient was set at MPH. MPH = (father's height + mother's height - 13)/2±4cm. Children with PAH or AH SDS ≥TH-1SDS were considered to have reached TH, while those with PAH or AH SDS <TH-1SDS were considered to have not reached TH. The following formula was used: PAH or FAH SDS = (PAH or AH-MPH)/5.4 (the final figure is obtained based on the year of the birth of the patient, with reference to the children data in the 1995 Nine-City Urban Census in China, the 1SDS for 15 year old is 5.5 cm). (5) Net height gain after GnRH treatment = FAH-PAH at the beginning of treatment, where net height gain reflects the long term effect of GnRHa treatment.(6) Calculation of Ht SDS -BA: Ht SDS -BA = (the actual height of patient-the 50th percentile height corresponding to BA)/SDS of this Ht-BA. (7) The percentage of patients who did not reach MPH is equal to the number of patients who did not reach the MPH/101×100%.

### Statistical analysis

Data were analyzed using SPSS 20.0 software package and expressed as mean ± viation. Patients who did not reach the MPH were expressed in percentage. Statistical analysis was conducted using t-test or paired t-test, One-way ANOVA, Chi-square test. Correlations between parameters were determined using Pearson correlation coefficient analysis. P<0.05 was considered as statistically significant.
